# Face matching in a long task: enforced rest and desk-switching cannot maintain identification accuracy

**DOI:** 10.7717/peerj.1184

**Published:** 2015-08-18

**Authors:** Hamood M. Alenezi, Markus Bindemann, Matthew C. Fysh, Robert A. Johnston

**Affiliations:** 1 School of Psychology, University of Kent, UK; 2 Department of Education and Psychology, Northern Borders University, KSA

**Keywords:** Face perception, Face matching, Unfamiliar faces, Passport control

## Abstract

In face matching, observers have to decide whether two photographs depict the same person or different people. This task is not only remarkably difficult but accuracy declines further during prolonged testing. The current study investigated whether this decline in long tasks can be eliminated with regular rest-breaks (Experiment 1) or room-switching (Experiment 2). Both experiments replicated the accuracy decline for long face-matching tasks and showed that this could not be eliminated with rest or room-switching. These findings suggest that person identification in applied settings, such as passport control, might be particularly error-prone due to the long and repetitive nature of the task. The experiments also show that it is difficult to counteract these problems.

## Introduction

In forensic face matching, observers have to decide whether two simultaneous presentations of unfamiliar faces depict the same person or different people (Jenkins & Burton, 2008; Johnston & Bindemann, 2013). This task is of considerable applied importance. Passport control at airports and national borders, for example, routinely requires the matching of a face photograph from an identity document to its bearer. Face matching is also utilized for person identification in other everyday settings, encompassing, for example, proof of age for the purchase of alcohol or as a method for controlling entrance to restricted premises.

A coherent body of research has established that face matching is a surprisingly error-prone task. Under optimized laboratory conditions, in which observers have to match pairs of high-quality same-day photographs, identification errors are typically made on 10–20% of trials (e.g., Burton, White & McNeill, 2010; Megreya, Bindemann & Havard, 2011). Identification accuracy declines further with more taxing task demands, for example, when to-be-matched photographs are taken months apart (Megreya, Sandford & Burton, 2013), viewing time is limited (Özbek & Bindemann, 2011), or realistic identity documents are used (Bindemann & Sandford, 2011; Kemp, Towell & Pike, 1997).

These identification errors might occur because face matching is image-bound (see Burton, Jenkins & Schweinberger, 2011; Jenkins & Burton, 2011; Jenkins et al., 2011). Changes in factors such as lighting, expression or view can induce many differences in the appearance of a face (for reviews, see Hancock, Bruce & Burton, 2000; Johnston & Edmonds, 2009). Every encounter with a person therefore provides a unique pattern for visual analysis. In the matching of unfamiliar faces, it can be difficult to dissociate the contribution of such variables to a person’s temporary appearance from the constant identity features of a face. As a consequence, two different people may seem to be the same person under similar viewing conditions, or two instances of the same person can be mistaken for separate people under different viewing conditions.

Such findings suggest that matching errors result from external factors that affect the appearance of a face. However, it is now becoming clear that internal factors, within observers, also contribute to errors in this task. Personality traits (Megreya & Bindemann, 2013), emotional states (Attwood et al., 2013), and an observer’s age (Megreya & Bindemann, 2015) can, for example, explain some variation *between* individuals in face-matching accuracy. However, there also appears to be variation *within* individuals in the ability to perform this task, as the same observers frequently make different identification decisions to repetitions of the same face pairs (Bindemann, Avetisyan & Rakow, 2012).

**Figure 1:**
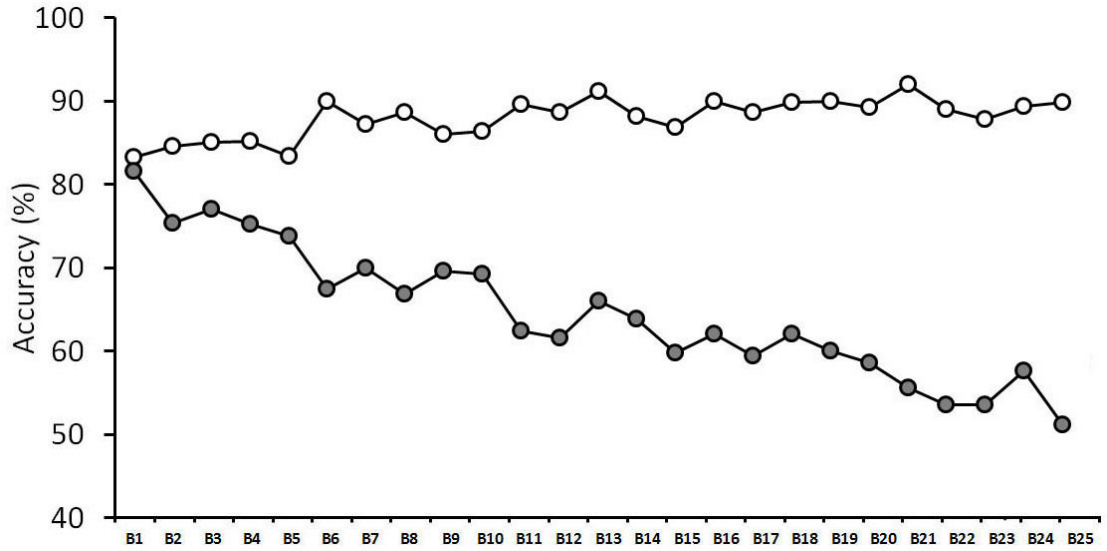
Face matching performance in a long task (taken from Alenezi & Bindemann, 2013). Open symbols denote match trials, grey-filled symbols denote mismatch trials. The data is split into 25 blocks of 40 trials, with each block comprising 20 match and 20 mismatch face pairs.

We recently obtained a striking example of this observer inconsistency (see Fig. 1). While face matching is typically measured with relatively short tasks, we assessed performance over 1,000 trials (Alenezi & Bindemann, 2013). Over this extended period, matching performance declined continuously. This effect was characterized by a remarkable error, whereby accuracy actually increased for identity matches, which are composed of two photographs of the same person’s face. However, accuracy decreased dramatically for mismatches, which consist of pairs of photographs of different people. This decline was such that mismatch accuracy was reduced to only 51% after 1,000 trials and showed no signs of reaching a floor level of performance.

These findings are important for several reasons. Firstly, this experiment indicates that general measures of matching accuracy, averaged over an entire experiment, might not be representative of performance at different stages of this task. Since the within-task variation in Alenezi & Bindemann’s (2013) study is characterized by a decline in accuracy over time, these findings suggest that previous research, which is based on shorter tasks and combines performance across blocks, might therefore still overestimate the accuracy of face matching. Moreover, the nature of this decline, which is characterized specifically by a decrease in mismatch accuracy, indicates that people find it increasingly difficult to tell different identities apart during prolonged face-matching tasks.

If these findings generalize to face-matching scenarios outside of the laboratory, then this error might be particularly serious for applied settings. Consider that passports are increasingly difficult to forge (e.g., National Audit Office, 2007; Bundesdruckerei, 2013). As a consequence, people who seek to cross borders without detection attempt to do so increasingly by using valid identity documents that belong to other persons who are of sufficiently similar facial appearance. These real-world identity mismatches are a documented security concern (e.g., CPNI, 2007; FRONTEX, 2012; Stevens, 2011). This concern seems justified considering that passport officers appear to be no better in face matching than the untrained student observers that are commonly used as participants in psychological experiments in this field (White et al., 2014b). In light of this, the finding that the detection of mismatches declines over prolonged laboratory testing raises further concerns about person identification at passport control, which is also a long and repetitive task.

The experiments in this study are motivated by this decline in mismatch accuracy in long tasks. We sought to explore two simple manipulations that were suggested to us by Border Force UK (J McSweeney, pers. comm., 2013) and might be useful for maintaining face-matching performance under such prolonged testing. Specifically, we explored whether accuracy could be maintained by enforcing regular rest-breaks (Experiment 1) or by asking observers to switch desks at regular intervals (Experiment 2).

## Experiment 1

This experiment sought to explore whether face-matching accuracy can be maintained in a long task by enforcing regular rest breaks. This makes good sense as breaks are a common form of resting the mind to maintain task performance. For this reason, rest breaks are also provided routinely in psychological experiments on face processing (see, e.g., Bonner & Burton, 2004; Farah et al., 1998; Özbek & Bindemann, 2011). However, it is unknown whether these breaks *actually* help to maintain performance in these tasks.

Outside of this domain, it has already been demonstrated that performance declines when breaks are not provided. Research has shown, for example, that observers find it difficult to sustain attention for continuous object identification and visual vigilance tasks (Davies & Parasuraman, 1982; Helton & Russell, 2012; Caggiano & Parasuraman, 2004). This reduction in accuracy becomes markedly pronounced after 20–30 min (Nuechterlein, Parasuraman & Jiang, 1983). Performance is also affected by monotony, whereby it is more challenging to maintain relevant task goals during highly repetitive tasks (see, e.g., Ariga & Lleras, 2011; Bonneh, Cooperman & Sagi, 2001; Helton & Russell, 2012). With regard to forensic face matching, it is already established that such decrements occur during related security tasks, such as baggage screening at airports (Nuechterlein, Parasuraman & Jiang, 1983). And outside of this field, it has been shown that compulsory rest breaks reduce errors in other applied tasks, such as surgical procedures (Engelmann et al., 2011). This raises the possibility that breaks can also help to maintain face-matching accuracy.

To assess whether such benefits can be found, Experiment 1 applied the same procedure as Alenezi & Bindemann (2013). The experiment therefore consisted of 1,000 trials of a matching task. However, to determine whether accuracy in this task can be maintained when participants are given regular rest periods, a five-minute break was enforced every 200 trials. In line with previous findings, we expected initial performance to be reasonably high and comparable for match and mismatch trials, followed by a continuous increase in match accuracy and a concurrent decline in mismatch performance (Alenezi & Bindemann, 2013). The question of main interest was whether breaks would facilitate recovery from this effect and re-set accuracy to its initial levels. To assess the effect of such rest breaks fully, performance was compared immediately prior to and after each break. In addition, we included a *control* condition for our analysis, to provide a direct comparison of face matching performance in this task when enforced rest-breaks are not administered. This comparison is based on Experiment 6 in Alenezi & Bindemann (2013) and provides identical sample size, stimuli and procedure to Experiment 1 (except for the enforced rest-breaks).

## Method

### Participants

Twenty-five undergraduate students (23 female) from the University of Kent, with a mean age of 22.9 years (*SD* = 8.9), volunteered to participate in the rest-break experiment for a small fee. The participants of the control group also comprised undergraduate volunteers (21 female) from the University of Kent, with a mean age of 20.0 years (*SD* = 2.9). All reported normal vision and provided written consent prior to taking part. This research was approved by the Ethics Committee of the School of Psychology at the University of Kent and was conducted in accordance with the ethical guidelines of the British Psychological Association.

### Stimuli and procedure

For the rest-break and the control group, the stimuli consisted of 100 match pairs (50 male, 50 female) and 100 mismatch pairs (also 50 male, 50 female) from the Glasgow Face Matching Test (see Burton, White & McNeill, 2010). These pairs were constructed so that faces were shown in grayscale on a white background. Each face was depicted with a neutral expression, and measured maximally 350 pixels in width at a screen resolution of 72 ppi. Only the internal features (i.e., the eyes, nose and mouth) of these faces were shown to minimize the influence of changeable external features, such as hairstyle (see, e.g., Bruce et al., 1999; Sinha & Poggio, 1996).

In each match and mismatch face pair, one face image was taken with a digital camera, while the other image was a frame from high-quality video. In addition, each pair consisted of a frontal and profile view. This change in view was retained for consistency with Alenezi & Bindemann (2013) and to reduce reliance on simple pictorial similarities between to-be-matched images (see, e.g., Jenkins & Burton, 2011). It also increases task difficulty (Estudillo & Bindemann, 2014; Longmore, Liu & Young, 2008). Considering that match responses increased continuously in Alenezi & Bindemann’s (2013) long face-matching task, this should reduce the possibility of ceiling effects.

### Procedure

Each trial began with a fixation cross for one second. This was followed by a face pair, which was presented until a response was registered. Participants were asked to decide whether an identity match or mismatch was shown by pressing one of two corresponding buttons on a computer keyboard. Accuracy was emphasized and responses were self-spaced.

The 200 face stimuli were administered in 5 blocks of 40 trials, comprising 20 match and 20 mismatch pairs. This sequence of blocks was then repeated four more times to provide a total of 1,000 trials across 25 experimental blocks. The presentation of the stimuli was randomized within all blocks for each observer. However, block order was counterbalanced across participants over the course of the experiment, so that each face pair was equally likely to appear in all of the blocks.

To enforce regular rest periods, participants were given a five-minute break every 200 trials (i.e., every five blocks). During these breaks, the onscreen display instructed participants to rest and entertainment magazines were provided to read. The provision of such magazines does not provide a rest for participants’ visual system *per se* but was designed to mimic possible break-time activities in occupational settings. A ten-second tone signalled the end of each rest period and alerted participants to return to the matching task. The procedure of the control group was identical except that these enforced rest-breaks between blocks were not provided.

## Results

### Accuracy

**Figure 2:**
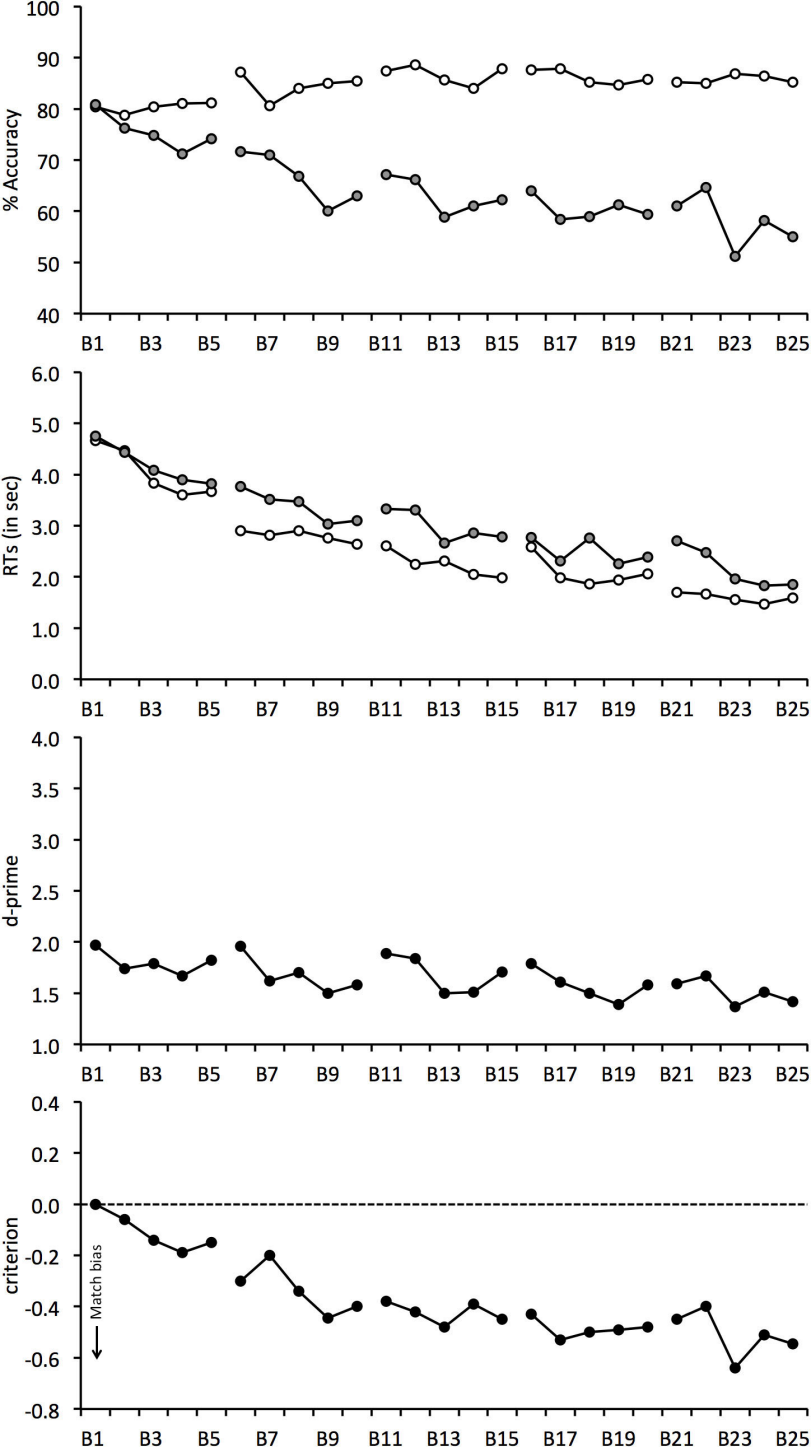
Face-matching performance for Experiment 1. The data is split into 25 blocks, illustrated on the horizontal axis. Individual graphs show percentage accuracy, response times, *d*-prime and criterion. Open symbols denote match trials and grey-filled symbols denote mismatch trials. Line breaks between blocks indicate enforced rest periods.

The percentage accuracy data are illustrated in Fig. 2 for each block of the experiment and show that performance was initially comparable for match and mismatch trials. Mismatch accuracy then began to decline and this effect persisted throughout the experiment. For match trials, on the other hand, a continuous increase in accuracy was observed.

To analyse these trends, match and mismatch accuracy were correlated with block number. This analysis shows a positive correlation for match trials, *r*(23) = 0.631, *p* < 0.01, but a negative correlation for mismatch trials, *r*(23) = − 0.871, *p* < 0.01. Figure 2 suggests that the continuous decline on mismatch trials is also more marked than the concurrent increase in accuracy on match trials. To explore this possibility, overall accuracy (i.e., the average of match and mismatch accuracy) was correlated with block. This revealed a negative correlation, *r*(23) = − 0.795, *p* < 0.01, which confirms that overall accuracy decreased during the experiment.

The correlational analysis indicates that enforced rest-breaks do not eliminate the decline in mismatch and overall accuracy that occurs during long matching tasks. To investigate this further, performance for the blocks immediately preceding and following each break was also analysed. For example, for the first 5-minute break this analysis compared performance on match and mismatch trials for Block 5 (before break) and Block 6 (after break). A 4 (break number: 1, 2, 3 and 4) × 2 (trial type: match vs. mismatch) × 2 (rest: before vs. after break) ANOVA revealed a main effect of trial type, *F*(1, 24) = 12.77, *p* < 0.01, ${\eta }_{p}^{2}=0.35$, which reflects generally lower accuracy for mismatch (*M* = 65.3%, *SD* = 22.5%) than match trials (*M* = 86.0%, *SD* = 13.4%). ANOVA also revealed a main effect of break number, *F*(3, 72) = 6.46, *p* < 0.01, ${\eta }_{p}^{2}=0.21$, due to the general decline in accuracy over the course of the experiment, with performance dropping from 78.6% (*SD* = 11.4%) at break 1 to 72.9% (*SD* = 13.4%) at break 4. In addition, an interaction between break number and trial type was found, *F*(3, 72) = 7.62, *p* < 0.01, ${\eta }_{p}^{2}=0.24$.

Analysis of simple main effects showed an effect of break number for mismatch trials, *F*(3, 72) = 5.97, *p* < 0.01, ${\eta }_{p}^{2}=0.45$, which reflects lower accuracy at breaks 2 (*M* = 65.1%, *SD* = 23.1%), 3 (*M* = 63.1%, *SD* = 25.2%) and 4 (*M* = 60.2%, *SD* = 27.7%) than at break 1 (*M* = 72.9%, *SD* = 17.4%), all *qs* ≥ 6.01, *ps* ≤ 0.01, and at break 4 than break 2, *q* = 3.77, *p* < 0.05. None of the other differences were significant, both *qs* ≤ 2.23. By contrast, accuracy was more even on match trials, *F*(3, 72) = 2.54, *p* = 0.08, ${\eta }_{p}^{2}=0.26$, with performance across breaks ranging only from 84.2% (*SD* = 14.5%) to 87.7% (*SD* = 12.9%). This analysis therefore converges with the correlational data to show that mismatch accuracy declined during the experiment. However, no main effect of rest, *F*(1, 24) = 2.18, *p* = 0.15, ${\eta }_{p}^{2}=0.08$, and no interaction of rest and break number, *F*(3, 72) = 0.68, *p* = 0.57, ${\eta }_{p}^{2}=0.03$, or rest and trial type, *F*(1, 24) = 0.04, *p* = 0.85, ${\eta }_{p}^{2}=0.00$, and no three-way interaction was found, *F*(3, 72) = 1.85, *p* = 0.15, ${\eta }_{p}^{2}=0.07$. This indicates, once again, that rest breaks do not alleviate the decline in mismatch accuracy.

### *d*-prime and criterion

The data were also transformed into signal detection measures, which reflect the combined accuracy on match and mismatch trials (*d*′) and response bias (criterion). These data are also given in Fig. 2 and show that overall accuracy (*d*′) decreased over the 25 blocks of the experiment, *r*(23) = − 0.659, *p* < 0.01. This decrease was accompanied by a criterion shift to classify increasingly more faces as identity matches over the course of the experiment, *r*(23) = − 0.878, *p* < 0.01.

To assess the effect of breaks on overall accuracy and bias, we also performed two 4 (break number: 1, 2, 3 and 4) × 2 (rest: before vs. after break) ANOVAs for *criterion* and *d*′. For *d*′, ANOVA did not find a main effect of rest, *F*(1, 24) = 2.72, *p* = 0.11, ${\eta }_{p}^{2}=0.10$, or an interaction of break number and rest, *F*(3, 72) = 1.17, *p* = 0.33, ${\eta }_{p}^{2}=0.05$. However, a main effect of break number was found, *F*(3, 72) = 3.73, *p* < 0.05, ${\eta }_{p}^{2}=0.14$. Tukey HSD test showed that this reflects lower accuracy at break 4 (*M* = 1.58, *SD* = 0.87) than break 1 (*M* = 1.89, *SD* = 0.85), *q* = 4.73, *p* < 0.01. The analogous analysis of *criterion* also did not find a main effect of rest, *F*(1, 24) = 0.08, *p* = 0.78, ${\eta }_{p}^{2}=0.00$, or an interaction between factors, *F*(3, 72) = 1.39, *p* = 0.25, ${\eta }_{p}^{2}=0.06$, but revealed a main effect of break, *F*(3, 57) = 5.59, *p* < 0.01, ${\eta }_{p}^{2}=0.19$. Tukey HSD test showed that *criterion* was lower at break 3 (*M* = − 0.44, *SD* = 0.56) and break 4 (*M* = − 0.46, *SD* = 0.63) in comparison with break 1 (*M* = − 0.23, *SD* = 0.41), both *qs* ≥ 4.71, *ps* ≤ 0.01. This bias confirms that observers made an increasingly greater proportion of match responses as the experiment progressed. None of the other comparisons were significant, all *qs* ≤ 3.58.

### Response times

Response speed was not emphasized to participants, but mean correct RTs were calculated also for completeness (see Fig. 2). For match and mismatch trials, response times decreased over the course of the experiment and correlated negatively with block number, *r*(23) = − 0.929, *p* < 0.01, and, *r*(23) = − 0.954, *p* < 0.01, respectively. In addition, a 4 (break number) × 2 (trial type) × 2 (rest) ANOVA of the RTs showed a main effect of trial type, *F*(1, 24) = 13.46, *p* < 0.01, ${\eta }_{p}^{2}=0.36$, due to generally longer RTs on mismatch (*M* = 3, 082 ms, *SD* = 1,597 ms) than match trials (*M* = 2, 519 ms, *SD* = 1,547 ms). A main effect of break number was also found, *F*(3, 72) = 6.26, *p* < 0.01, ${\eta }_{p}^{2}=0.21$, which reflects the decrease in response times over the course of the experiment, ranging from 3,538 ms (*SD* = 2, 325) at break 1 to 2,213 ms (*SD* = 1, 308) at break 4. However, no main effect of rest was found, *F*(1, 24) = 0.00, *p* = 0.96, ${\eta }_{p}^{2}=0.00$. None of the two-way interactions, all *Fs* ≤ 1.05, *ps* ≥ 0.32, ${\eta }_{p}^{2}\leq 0.04$, or the three-way interaction, *F*(3, 57) = 2.00, *p* = 0.12, ${\eta }_{p}^{2}=0.07$, were significant.

### Enforced-rest vs. control condition

In a final step of the analysis, performance with enforced rest-breaks was compared with a control condition, in which such breaks were not provided (for a summary of the control data, see Fig. 1). A 4 (break number: 1, 2, 3 and 4) × 2 (trial type: match vs. mismatch) × 2 (rest: before vs. after break) × 2 (condition: enforced rest vs. control) mixed-factor ANOVA found no main effect of condition, *F*(1,48) = 0.02, *p* = 0.89, ${\eta }_{p}^{2}=0.00$, and no interactions between condition and any of the other factors, all *Fs* ≤ 3.40, *ps* ≥ 0.07, ${\eta }_{p}^{2}\leq 0.07$.

## Discussion

Identification performance was initially relatively high, at just over 80%, for match and mismatch trials. Thereafter, performance declined on mismatch trials throughout the experiment. This decline was such that mismatch accuracy dropped close to 50% in Block 25 and was accompanied by a concurrent increase in match responses. This pattern indicates that the face pairs, regardless of stimulus type, were classified increasingly as identity matches. This bias suggests that observers lost the ability to tell different facial identities apart during this long task.

These results converge with Alenezi & Bindemann’s (2013) recent findings. In contrast to this previous work, the current experiment explored whether enforced rest periods of five minutes could help to maintain face-matching accuracy. We failed to find evidence of such a benefit here, both within the rest-break condition and in comparison with a control group for which such rests were not provided. This is striking considering the duration of these rest periods compared to the time that observers required to complete the intervening face-matching trials. For example, whereas Blocks 1 to 5 were completed on average in 17.1 min, by Blocks 21–25 this had reduced to only 9.6 min. By the end of the experiment, observers therefore received one minute of rest for every two minutes of the matching task. This favourable ratio suggests that matching performance does not decline because of insufficient rest time. In turn, these findings indicate that rest breaks are not effective for maintaining matching performance.

## Experiment 2

Experiment 1 showed that enforced breaks do not help to maintain performance in a long face-matching task. The next experiment therefore sought to explore an alternative manipulation that could be employed in applied settings with minimal effort, by moving observers into a new room after every five blocks. We compare this subtle change in context to a desk-switching exercise whereby passport officers in settings such as an airport arrivals hall would be asked to interchange control points at regular intervals.

This manipulation is explored here due to its simple practical potential but it also receives some support from psychological theory. It is well established, for example, that the maintenance of contextual cues facilitates recognition memory for a range of visual stimuli (e.g., Rosas et al., 2001; Smith & Vela, 2001; Godden & Baddeley, 1980), including unfamiliar faces (Russo et al., 1999). If *poor* performance is also maintained by the continuation of the same context, then a decline in accuracy might be alleviated by change.

This experiment is motivated also by the fact that observers typically exhibit the best face-matching accuracy at the start of an experiment, whereas performance deteriorates as the task progresses (Alenezi & Bindemann, 2013). This pattern might arise from habituation, whereby participants struggle to maintain the same goal representations over time due to the repetitive nature of this task. Such habituation can be alleviated through the momentary de- and reactivation of a task (Ariga & Lleras, 2011; Helton & Russell, 2012; Bonneh, Cooperman & Sagi, 2001). Room-switching might also help to prevent habituation  in this way. If this approach is successful, then it should maintain matching accuracy.

## Method

### Participants

Twenty-five new students (21 female) from the University of Kent, with a mean age of 20.3 years (*SD* = 2.6), participated in the room-switching experiment for a small fee. All reported normal vision and provided written consent prior to taking part. This research was approved by the Ethics Committee of the School of Psychology at the University of Kent and was conducted in accordance with the ethical guidelines of the British Psychological Association.

### Stimuli and procedure

The stimuli and procedure were identical to Experiment 1, except for the following changes. The experiment took part in a laboratory, which comprised a waiting area and five testing booths. During the experiment, participants again performed 1,000 trials of the matching task, comprising 25 blocks of 20 match and 20 mismatch trials. However, in contrast to the rest breaks of Experiment 1, participants now changed rooms regularly, by moving into a new experimental booth after every 200 trials. Each of these changeovers required less than one minute.

## Results

### Accuracy

**Figure 3:**
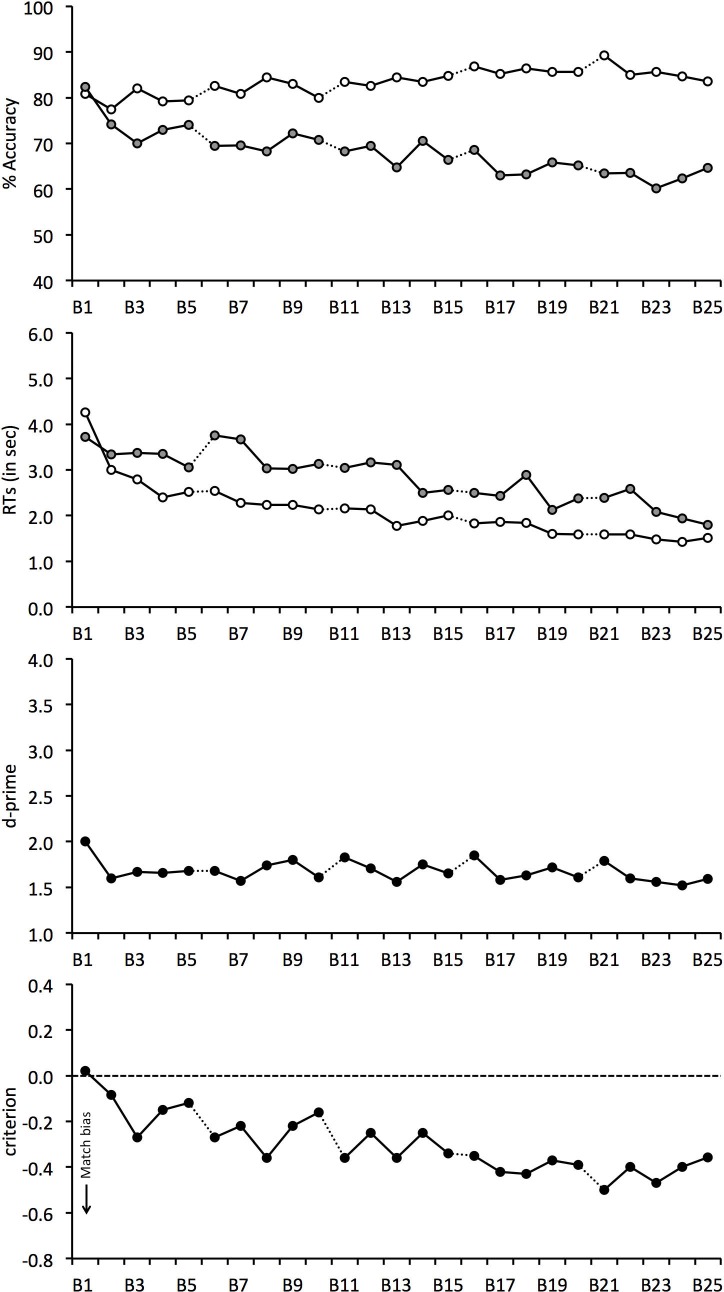
Face-matching performance for Experiment 2. The data is split into 25 blocks, illustrated on the horizontal axis. Individual graphs show percentage accuracy, response times, *d*-prime and criterion. Open symbols denote match trials and grey-filled symbols denote mismatch trials. Dotted lines between blocks indicate room switches.

The mean percentage accuracy for match and mismatch trials is shown in Fig. 3. A correlation of these scores with block number revealed a positive relationship on match trials, *r*(23) = 0.773, *p* < 0.001, which shows that match responses gradually increased during the experiment. The opposite pattern was found for mismatch trials, for which accuracy declined continuously, *r*(23) = − 0.854, *p* < 0.001. As in Experiment 1, overall accuracy was also analysed. This revealed a negative correlation, *r*(23) = − 0.607, *p* < 0.01, which shows that overall performance decreased during the experiment.

The correlational analysis indicates that room-switching does not eliminate the decline in mismatch accuracy during long matching tasks. To investigate this further, matching performance before and after each of the room-switches was also compared. A 4 (switch number: 1, 2, 3 and 4) × 2 (trial type: match vs. mismatch) × 2 (room switch: before vs. after) ANOVA of these data showed a main effect of trial type, *F*(1, 24) = 10.86, *p* < 0.01, ${\eta }_{p}^{2}=0.31$, due to generally lower accuracy on mismatch (*M* = 68.3%, *SD* = 14.3%) than match trials (*M* = 84.0%, *SD* = 15.5%). However, main effects of room switch, *F*(1, 24) = 0.23, *p* = 0.64, ${\eta }_{p}^{2}=0.01$, and switch number, *F*(3, 72) = 0.32, *p* = 0.81, ${\eta }_{p}^{2}=0.01$, were not found.

The interaction of switch number and room switch, *F*(3, 72) = 0.71, *p* = 0.55, ${\eta }_{p}^{2}=0.03$, and room switch and trial type, *F*(1, 24) = 3.57, *p* = 0.07, ${\eta }_{p}^{2}=0.13$, and the three-way interaction, *F*(3, 72) = 0.89, *p* = 0.45, ${\eta }_{p}^{2}=0.04$, were not significant. However, an interaction between switch number and trial type was found, *F*(3, 72) = 8.06, *p* < 0.001, ${\eta }_{p}^{2}=0.25$. Analysis of simple main effects shows that performance declined on mismatch trials, *F*(3, 72) = 3.66, *p* < 0.05, ${\eta }_{p}^{2}=0.33$, so that accuracy at the fourth switch (*M* = 64.3%, *SD* = 16.1%) was lower than at the first (*M* = 71.7%, *SD* = 14.6%) and second switch (*M* = 69.5%, *SD* = 15.5%), both *qs* ≥ 4.37, *ps* ≤ 0.05. Analysis of simple main effects also showed a change in performance in the match condition, *F*(3, 72) = 7.28, *p* < 0.001, ${\eta }_{p}^{2}=0.50$, due to an increase in accuracy by switch 4 (*M* = 87.4%, *SD* = 12.9%) compared to switch 1 (*M* = 81.0%, *SD* = 17.6%) and 2 (*M* = 81.7%, *SD* = 18.6%), both *qs* ≥ 4.79, *p* ≤ 0.05, and switch 3 (*M* = 85.8%, *SD* = 15.0%) compared to switch 1, *q* = 4.03, *p* < 0.05. None of the other differences were significant, all *qs* ≤ 3.53. In addition, a simple main effect of trial type was not found at switch 1 (match *M* = 81.0%, *SD* = 17.6%; mismatch *M* = 71.7%, *SD* = 14.5%), *F*(1, 24) = 3.38, *p* = 0.08, ${\eta }_{p}^{2}=0.12$, but at switch 2 (match *M* = 81.7%, *SD* = 18.6%; mismatch *M* = 69.5%, *SD* = 15.5%), *F*(1, 24) = 5.07, *p* < 0.05, ${\eta }_{p}^{2}=0.17$, switch 3 (match *M* = 85.8%, *SD* = 15.0%; mismatch *M* = 67.5%, *SD* = 17.5%), *F*(1, 24) = 10.58, *p* < 0.01, ${\eta }_{p}^{2}=0.31$, and at switch 4 (match *M* = 87.4%, *SD* = 12.9%; mismatch *M* = 64.3%, *SD* = 16.1%), *F*(1, 24) = 28.48, *p* < 0.001, ${\eta }_{p}^{2}=0.54$, due to higher accuracy on match than mismatch trials.

### *d*-prime and criterion

The accuracy data were again transformed into the signal detection measures of *d*′ prime and criterion (Fig. 3). *d*′ scores declined throughout the task, due to the gradual decrease in overall accuracy during the experiment, and correlated negatively with block, *r*(24) = − 0.360, *p* = 0.075. For criterion, accuracy was initially close to zero score, which indicates that observers were equally likely to make correct match or mismatch responses at the beginning of the experiment. Criterion then began to decrease immediately after Block 1 and this decline continued throughout the matching task, *r*(24) = − 0.826, *p* < .001. This reflects a growing bias to make more match responses over the course of the experiment.

We also performed two 4 (switch number: 1, 2, 3 and 4) × 2 (room switch: before vs. after switch) ANOVAs for *criterion* and *d*′. For *d*′, ANOVA did not find a main effect of switch number, *F*(3, 72) = 0.23, *p* = 0.87, ${\eta }_{p}^{2}=0.01$, room switch, *F*(1, 24) = 1.72, *p* = 0.20, ${\eta }_{p}^{2}=0.07$, or an interaction between these factors, *F*(3, 72) = 0.54, *p* = 0.66, ${\eta }_{p}^{2}=0.02$. The analogous analysis of *criterion* also did not find an interaction between factors, *F*(3, 72) = 1.39, *p* = 0.25, ${\eta }_{p}^{2}=0.06$, but revealed a main effect of room switch, *F*(1, 24) = 6.72, *p* < 0.05, ${\eta }_{p}^{2}=0.22$, which reflects a greater match bias after (*M* = − 0.37, *SD* = 0.44) than before each switch (*M* = − 0.25, *SD* = 0.40). A main effect of switch number was also found, *F*(3, 72) = 7.05, *p* < .001, ${\eta }_{p}^{2}=0.23$. Tukey HSD test showed that *criterion* was lower at the fourth (*M* = − 0.44, *SD* = 0.41) in comparison with the first (*M* = − 0.20, *SD* = 0.44) and second room-switch (*M* = − 0.26, *SD* = 0.40), both *qs* ≥ 4.17, *ps* ≤ 0.05. This bias confirms that observers made an increasingly greater proportion of match responses as the experiment progressed. None of the other comparisons were significant, all *qs* ≤ 3.68.

### Response times

For completeness, Fig. 3 also shows the mean correct RTs. For match and mismatch trials, these data correlated negatively with block, *r*(23) = − 0.864, *p* < 0.001 and *r*(23) = − 0.904, *p* < 0.001, respectively. This shows that response speed increased during the experiment. A 4 (switch number) × 2 (trial type) × 2 (room switch) ANOVA of these data also showed a main effect of switch number, *F*(3, 72) = 8.75, *p* < 0.001, ${\eta }_{p}^{2}=0.27$, due to the decline in response times during the experiment, which ranged from 2,966 ms (*SD* = 1,536 ms) at switch 1 to 1,989 ms (*SD* = 783 ms) at switch 4. In addition, a main effect of trial type was found, *F*(1, 24) = 16.15, *p* < 0.001, ${\eta }_{p}^{2}=0.40$, reflecting generally longer RTs on mismatch (*M* = 2, 851 ms, *SD* = 1, 524 ms) than match trials (*M* = 2,046 ms, *SD* = 921 ms). By contrast, no main effect of room switch was found, *F*(1, 24) = 0.26, *p* = 0.62, ${\eta }_{p}^{2}=0.01$, and all two-way interactions, all *Fs* ≤ 1.53, *ps* ≥ 0.21, ${\eta }_{p}^{2}\leq 0.06$, and the three-way interaction, *F*(3, 72) = 1.19, *p* = 0.32, ${\eta }_{p}^{2}=0.05$, were not significant.

### Room-switching vs. control condition and rest-breaks

In a final step of the analysis, performance with room switches was also compared with the control condition (see Experiment 1), in which such breaks were not provided (for a summary of the control data, see Fig. 1). A 4 (switch number: 1, 2, 3 and 4) × 2 (trial type: match vs. mismatch) × 2 (room switch: before vs. after switch) × 2 (condition: room switch vs. control) mixed-factor ANOVA found no main effect of condition, *F*(1, 48) = 0.02, *p* = 0.96, ${\eta }_{p}^{2}=0.00$, and no interactions between condition and any of the factors, all *Fs* ≤ 1.95, *ps* ≥ 0.17, ${\eta }_{p}^{2}\leq 0.04$, except for an interaction of condition and switch number, *F*(3, 144) = 3.14, *p* < 0.05, ${\eta }_{p}^{2}=0.06$. Analysis of simple main effects found no effect of switch number for the room-switching condition, *F*(3, 144) = 0.37, *p* = 0.77, ${\eta }_{p}^{2}=0.02$, but for the control condition, *F*(3, 144) = 4.23, *p* < 0.05, ${\eta }_{p}^{2}=0.22$. Tukey HSD test showed that this arises from lower accuracy at switch 3 (*M* = 74.7%, *SD* = 7.9%) and 4 (*M* = 73.9%, *SD* = 9.3%), than switch 1 (*M* = 78.7%, SD = 8.5%), both *qs* ≥ 4.49, both *ps* ≤ 0.05. This contrast between the experimental conditions could indicate that room-switches slow the decline in accuracy that occurs during the course of the experiment. Contrary to this notion, however, no simple main effects of condition were found for any of the four individual room switches, all *Fs* ≤ 0.91, all *ps* ≥ 0.35, all ${\eta }_{p}^{2}\leq 0.02$.

A similar analysis was also conducted to compare enforced rest-breaks and room switches directly. A 4 (break /switch number: 1, 2, 3 and 4) × 2 (trial type: match vs. mismatch) × 2 (rest/switch: before vs. after) × 2 (condition: rest break vs. room switch) mixed-factor ANOVA found no main effect of condition, *F*(1, 48) = 0.03, *p* = 0.87, ${\eta }_{p}^{2}=0.00$, and no interactions between condition and any of the factors, all *Fs* ≤ 1.23, *ps* ≥ 0.27, ${\eta }_{p}^{2}\leq 0.03$, except for an interaction of condition and rest/switch number, *F*(3, 144) = 3.12, *p* < 0.05, ${\eta }_{p}^{2}=0.06$. Analysis of simple main effects found no effect of rest/switch number for the room-switching condition, *F*(3, 144) = 0.34, *p* = 0.79, ${\eta }_{p}^{2}=0.02$, but for the rest-break condition, *F*(3, 144) = 5.75, *p* < 0.01, ${\eta }_{p}^{2}=0.27$. Tukey HSD test showed that this arises from lower accuracy at break 4 (*M* = 72.9%, *SD* = 13.4%) than break 1 (*M* = 78.6%, *SD* = 11.4%), *q* = 6.46, *p* < 0.05. However, no simple main effects of condition were found for any of the four individual room switches, all *Fs* ≤ 0.82, all *ps* ≥ .37, all ${\eta }_{p}^{2}\leq 0.02$.

## Discussion

As in Experiment 1, matching accuracy was initially comparatively high but then declined throughout the experiment. This effect was characterised by a decline in mismatch accuracy, whereas performance for match trials showed a concurrent increase in accuracy but of a lesser magnitude. This pattern replicates the response bias from previous studies (Alenezi & Bindemann, 2013) and suggests that observers find it increasingly difficult to tell different identities apart in prolonged face-matching tasks.

Experiment 2 examined whether this bias is reduced when observers switch rooms at regular intervals. A visual comparison with a control group and the enforced-rest condition suggests that room-switches might have slowed the decline in mismatch accuracy to some extent (c.f., Figs. 1–3). However, this observation receives only limited support from the statistical analysis. Moreover, the results clearly show that room-switching cannot *eliminate* the decline in mismatch, and overall, accuracy that is found in long face-matching tasks.

It is possible that room-switches failed to affect matching performance because the experimental booths were visually highly similar spaces. We specifically chose this set-up in view of the practical implications of our work, such as person identification at passport control, where desk-switching would expose operators to comparable similarities across work-stations. Consequently, the current data suggests that desk-switching would not be effective for maintaining face-matching accuracy at passport control.

## General Discussion

It is well established that face matching is an error-prone task (see, e.g., Burton, White & McNeill, 2010; Johnston & Bindemann, 2013). However, it has only emerged recently that matching performance declines further during prolonged testing (Alenezi & Bindemann, 2013). This decline in accuracy is characterized by a striking error, whereby accuracy increases for identity matches but decreases for mismatches. This pattern indicates that observers find it increasingly difficult to tell different people apart under such conditions.

The current study explored two manipulations that might help to arrest this decline in mismatch accuracy, by investigating the effect of rest-breaks and room-switching on task performance. These manipulations were chosen due to their simple practical application, but also receive support from psychological theory. It has already been shown, for example, that it is difficult to sustain attention for long and monotonous tasks (see, e.g., Caggiano & Parasuraman, 2004; Helton & Russell, 2012; Nuechterlein, Parasuraman & Jiang, 1983) and that rest breaks can reduce errors in some applied tasks (Engelmann et al., 2011). Similarly, there is evidence that the momentary de- and reactivation of task-goals can improve visual performance (see, e.g., Ariga & Lleras, 2011; Bonneh, Cooperman & Sagi, 2001; Helton & Russell, 2012). However, it has not been explored whether such manipulations benefit facial identification.

In both experiments here, accuracy began to decline after the first block of trials. This effect persisted throughout the experiment and, consistent with previous research, was characterised by a specific decline in mismatch accuracy (c.f., Alenezi & Bindemann, 2013). However, this effect could not be eliminated by regular rest-breaks and room-switching. This is striking considering the magnitude of these manipulations. For example, towards the end of Experiment 1, observers received one min of rest for every two minutes of the task. Despite this favourable task-to-break ratio, no improvements in accuracy were found. Taken together, these results indicate that enforced rest-breaks and room-switching are not effective at maintaining face-matching accuracy. This also suggests that the decline in mismatch accuracy cannot be attributed *per* se to mental fatigue or the habituation of task goals. We draw these conclusions with some caution considering current sample sizes (with *N* = 25 per condition). It is possible that effects of rest-breaks and room-switching are still found with larger samples or different participant groups, such as passport officers (but see White et al., 2014b).

## Practical Application

This study provides further evidence that the difficulty of face matching might be underestimated by experiments that assess performance over a short duration (see Alenezi & Bindemann, 2013; Bindemann, Avetisyan & Rakow, 2012). These difficulties are likely to apply to important security tasks, such as passport control (see White et al., 2014b), where faces have to be matched continuously over long intervals. The current experiments show that it is difficult to counteract the loss of accuracy that occurs during such prolonged face matching tasks with rest-breaks or room-switching. Solutions to this problem in applied settings may therefore require alternative approaches to improve the accuracy of human observers, such as the redesign of current photo-identity documents (White et al., 2014a) or crowd-based decision-making (White et al., 2013).

Our findings could also inform how technological solutions to person identification at passport control, such as electronic passport gates (eGates), should be combined best with human operators to maximise performance. These eGates utilise facial recognition software to automatically compare a person’s face to their passport photograph and are now used across many countries. Doubts remain, however, over the accuracy of these systems (see Jenkins & White, 2009; Robertson, Middleton & Burton, 2015). In applied settings (and our experiments), both face matches and mismatches present two different images of a person. Electronic recognition systems must, therefore, have a threshold to determine when sufficient similarity exists between images to make identification decisions. The current findings suggest that it might be advantageous to apply a threshold to electronic recognition systems that is biased towards mismatch responses. This might help to counteract the match bias of human observers, which are typically used to verify electronically-detected mismatches. However, such counterbalancing is complicated considering the match bias that arises gradually in human observers during prolonged testing, whereas electronic recognition thresholds are stable. The combination of these processes is therefore an interesting area for further research.

## Supplemental Information

10.7717/peerj.1184/supp-1 Supplemental Information 1 Raw Data for Experiment 1 and 2
